# Effects of Postoperative Atropine Eye Drops on Visual Quality in Patients Undergoing Trabeculectomy

**DOI:** 10.3390/jcm12030763

**Published:** 2023-01-18

**Authors:** Panagiotis Laspas, Elisa Maier, Alexander Schuster, Colm McAlinden, Norbert Pfeiffer, Esther Hoffmann

**Affiliations:** 1 Department of Ophthalmology, University Medical Center, Johannes Gutenberg-University Mainz, Langenbeckstr. 1, 55131 Mainz, Germany; 2 Corneo Plastic Unit & Eye Bank, Queen Victoria Hospital, East Grinstead RH19 3DZ, UK; 3 Wenzhou Medical University, Wenzhou, China; 4 Eye & ENT Hospital of Fudan University, Shanghai, China

**Keywords:** atropine, eye drops, trabeculectomy, visual quality

## Abstract

The aim of this study was to investigate the effects of atropine on patients’ perception of visual quality after trabeculectomy. Forty patients undergoing standard trabeculectomy with mitomycin C were enrolled in this prospective randomized study. All surgeries were conducted at the ophthalmology department of the University Medical Center of Mainz, Germany. All patients received the same postoperative treatment with ofloxacin and dexamethasone eye drops. Following randomization of patients into two groups of 20 patients, the intervention group also received atropine eye drops three times daily for the first 2 days after surgery to stabilize the anterior chamber. All patients completed a visual quality questionnaire before surgery and 2 and 6 weeks after surgery. Results were compared using the Wilcoxon test. After surgery, there was a reduction in visual quality in all patients. Patients who received atropine eye drops described a greater and longer-lasting reduction in visual quality than those who did not receive atropine eye drops. Trabeculectomy often leads to a transient reduction in visual quality. This reduction was greater in severity and duration in patients who received postoperative atropine eye drops. Thus, unless there is an underlying medical necessity, we would discourage the application of atropine as a standard therapy for trabeculectomy surgery.

## 1. Introduction

Trabeculectomy is currently the gold-standard surgical treatment for glaucoma [1]. The objective of this procedure is to establish an outflow of aqueous humor from the anterior chamber into the subconjunctival space to reduce and control intraocular pressure [2]. A potentially dangerous complication of trabeculectomy is early hyperfiltration with hypotony and flattening of the anterior chamber [3]; this can lead to choroidal effusion [4], suprachoroidal bleeding [5], and secondary aqueous misdirection syndrome [6].

Atropine is a non-selective muscarinic receptor antagonist commonly prescribed after trabeculectomy for its cycloplegic properties [7]. It induces relaxation of the ciliary muscles, tightening the lens zonules. This results in a posterior displacement of the iris–lens diaphragm and deepening of the anterior chamber [8]. Another beneficial postoperative effect is stabilization of the blood–aqueous barrier, reducing inflammation in the anterior chamber [9].

However, atropine also has mydriatic properties that can cause multiple visual side effects [10], including disruption of accommodation, blurred vision, perception of haloes, and light sensitivity. The potential systemic absorption of atropine can also have unintended side effects on the cardiovascular system [11], such as dysrhythmias, as well as on patient mental state, causing confusion, restlessness, and emotional lability [12].

Thus, the necessity of postoperative treatment with atropine eye drops after trabeculectomy has been called into question. Comparative studies on biometric changes have already been conducted [13]. Therefore, our study was focused on the visual side effects of atropine following trabeculectomy, with particular focus on patient perception of visual quality.

## 2. Materials and Methods

### 2.1. Patient Selection and Study Design

This prospective, double-blind, randomized study included 40 patients who underwent trabeculectomy for glaucoma. The surgical procedure and possible postoperative treatment regimens were fully explained to patients before they provided written informed consent. Subsequently, study participants were randomly enrolled into two groups determined by a computer program. A total of 20 patients in the intervention group received atropine eye drops as part of their postoperative care plan, whereas 20 patients in the control group did not receive postoperative atropine eye drops.

Study approval was provided by the ethics committee of Rhineland-Palatinate, and all procedures conformed to the tenets of the Declaration of Helsinki. All ongoing trials for this intervention have been registered.

The inclusion criteria were planned primary trabeculectomy in cases of primary or secondary open-angle glaucoma or normal-pressure glaucoma. Exclusion criteria were any contraindications for the application of atropine.

### 2.2. Trabeculectomy

All patients in this study underwent a standard fornix-based trabeculectomy at the Department of Ophthalmology of the University Medical Center of Mainz, Germany [14]. All patients received a standardized follow-up assessment with their surgeon on the first day following surgery. Antimetabolite application (subconjunctival injections of 5-fluorouracil), suture lysis, and bleb massage were performed after careful evaluation of the appearance and filtration of the bleb. Postoperative treatment for all patients included dexamethasone eye drops (1.3 mg/mL) three times daily and ofloxacin eye drops (3 mg/mL) four times daily. Patients in the atropine group were also treated with atropine eye drops (1 mg/mL) three times daily for 2 days, whereas patients in the control group were not.

### 2.3. Questionnaire

As our goal was to investigate the effects of atropine on our patients’ subjective perceptions of visual impairment after trabeculectomy, we did not perform comparisons of numerical values such as visual acuity. Furthermore, personal perception of and satisfaction with quality of vision can vary among patients with similar objective visual measurements. A questionnaire assessing possible disturbances in visual function was created in German, similar to other available questionnaires [15]. Questions addressed the ten following visual disturbances: light sensitivity, haloes, starbursts, hazy vision, blurred vision, distortion, double vision, unstable vision, difficulty in focusing, and difficulty of estimating distances or depth. As in previous studies, each disturbance type was assessed with respect to frequency, severity, and bothersomeness. Furthermore, an initial question regarding was included eliciting a general rating of visual quality. Thus, altogether the questionnaire comprised 31 questions (1 + 3 × 10).

### 2.4. Statistical Analysis

The Wilcoxon test (IBM Corp. Released 2019. IBM SPSS Statistics for Windows, Version 26.0. Armonk, NY, USA: IBM Corp ) was used for comparisons between the different time points within the study groups. Statistical significance was set at *p* < 0.05.

## 3. Results

### 3.1. Patient Demographics

This study included 18 men and 22 women. The mean age of patients in the atropine group was 69.50 ± 8.63 years and 68.15 ± 13.75 years in the control group (*p* > 0.05; Mann–Whitney U test). In the atropine group, 13 patients had open-angle glaucoma, 5 had pseudoexfoliative glaucoma, 1 had angle-closure glaucoma, and 1 had normal-pressure glaucoma. In the control group, there were 11 patients with open-angle glaucoma, 4 patients with pseudoexfoliative glaucoma, 3 patients with normal-pressure glaucoma, 1 patient with secondary glaucoma, and 1 patient with late-juvenile glaucoma.

### 3.2. Atropine Group

**Figure 1:**
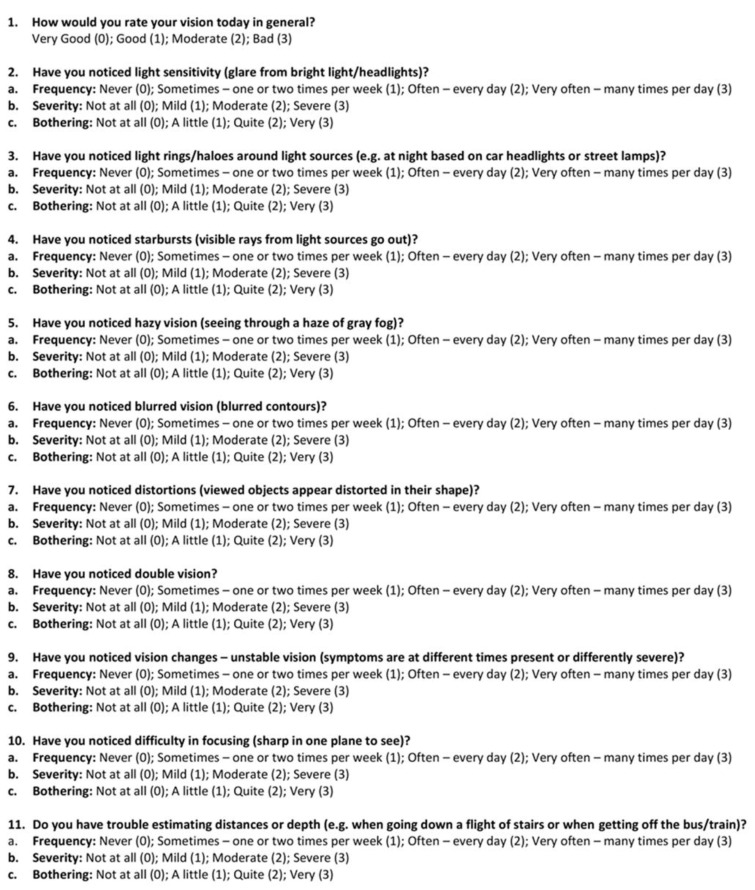
Questionnaire of visual quality. The primary question targeted the perception of the overall visual quality. The following 10 questions addressed visual disturbances that are common in patients receiving atropine eye drops: light sensitivity, haloes, starbursts, hazy vision, blurred vision, distortion, double vision, unstable vision, difficulty in focusing, and difficulty in estimating distances or depth. Each of these 10 questions was answered in relevance to frequency, severity, and bothersomeness. Participants answered each question on a scale from 0 (least frequent, severe, or bothersome) to 3 (most frequent, severe, or bothersome).

**Figure 2:**
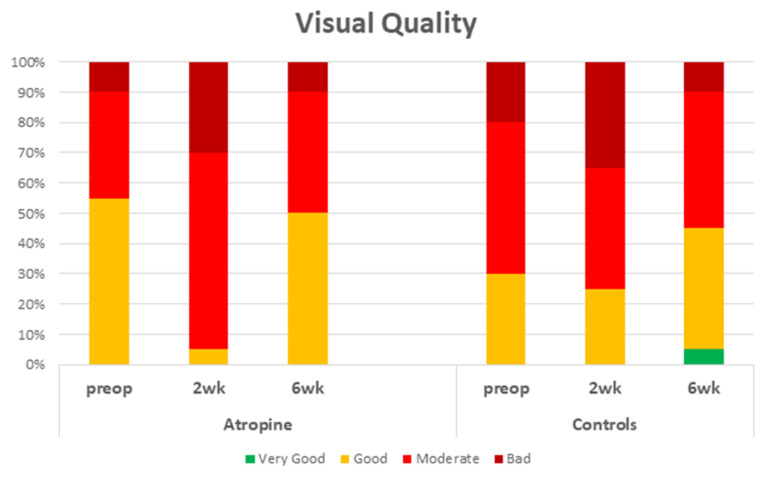
Overall visual quality in both groups. The percentage of patients given each possible value is presented. In the atropine, group there is an obvious reduction in visual quality reported two weeks after surgery compared with the preoperative evaluation. In the control group, there is no significant reduction in the visual quality. Six weeks after surgery, both groups report visual quality comparable to their preoperative status.

Two weeks after surgery, patients in the atropine group had a significant reduction (Z/P = 0.00453) in their self-assessed overall visual quality compared with their preoperative assessments (Figure 1). The percentage of patients reporting moderate or poor visual quality jumped from 45% to 95%. Six weeks after surgery, this percentage was almost fully corrected to 50% (W/P < 0.05). Concerning the different qualitative aspects of vision, two weeks after surgery, patients in the atropine group had more frequent, severe, and bothersome hazy vision (Z/P = 0.00256; W/P < 0.05; W/P < 0.05), blurred vision (Z/P = 0.00015; Z/P = 0.00048; Z/P = 0.00074), and difficulties in estimating distances or depth (Z/P = 0.0139; Z/P = 0.0139; Z/P = 0.00798). Moreover, they reported more frequent and more distressing sensitivity to light (Z/P = 0.03438; Z/P = 0.02275) and perception of haloes (Z/P = 0.02068; Z/P = 0.03673). They also had more frequent difficulties in focusing (Z/P = 0.03005). Six weeks after surgery, they reported fewer visual problems, but they still complained of more frequent and more distressing starbursts (Z/P = 0.02068; W/P < 0.05) and blurred vision (Z/P = 0.02222; Z/P < 0.04182) compared with their preoperative status. The percentage of patients who reported having often or always blurred vision climbed from 20% to 80% at the end of the second postoperative week and remained significantly high (65%) at the end of the sixth postoperative week (Figure 2). Compared to only 20% preoperatively, 75% of the patients in the atropine group assessed blurred vision as quite bothersome or very bothersome two weeks postoperatively. Six weeks postoperatively, this percentage was not completely corrected and remained significantly high at 40%.

### 3.3. Control Group

Two weeks after surgery, patients in the control group reported similar overall visual quality to their preoperative evaluation (Z/P = 0,18406). Six weeks after surgery, patients in the control group once again showed no differences (Z/P = 0.07078) in their scores concerning their overall assessment (Figure 1). The percentage of patients reporting a moderate or poor visual quality increased slightly from 70% to 75% two weeks after surgery and was more than fully corrected (55%) six weeks after surgery. On the other hand, two weeks after surgery, they specifically reported more frequent, severe, and bothersome haze (Z/P = 0.00336; Z/P = 0.00379; Z/P = 0.00964) and blurred vision (Z/P = 0.00048; Z/P = 0.00298; Z/P = 0.00298) than they did preoperatively based on their self-assessment. This group also experienced more frequent problems with focusing (Z/P = 0.03438). Six weeks after surgery, all different aspects of visual quality were evaluated similarly to their preoperative assessments. For example, the percentage of patients in the control group who reported having often or always blurred vision climbed from 30% to 55% at the end of the second postoperative week but was almost fully corrected to 35% at the end of the sixth postoperative week (Figure 2). The percentage of patients who evaluated blurred vision as quite bothersome or very bothersome increased from 40% to 65% two weeks postoperatively, but this was corrected completely to 40% six weeks postoperatively.

## 4. Discussion

Our study reveals some interesting findings concerning early postoperative management strategies for patients following trabeculectomy and their effects on self-reported visual quality. Our primary area of interest was how atropine eye drops affect visual quality and the rehabilitation process in post-trabeculectomy patients. Atropine eye drops remain a standard postoperative medication for trabeculectomy; however, their visual and systemic side effects may outweigh their benefits.

Orengo-Nania et al. were the first to evaluate the biometrical and other effects of atropine eye drops following trabeculectomy in a randomized and prospective study [13]; however, it could not be confirmed whether atropine eye drops caused a reduction in intraocular inflammation or deepening of the anterior chamber. The study suggested that from an anatomical point of view, there is no hard evidence that the standard usage of atropine eye drops is necessary.

On the other hand, in trabeculectomy cases with shallow anterior chambers, atropine eye drops may help to deepen the anterior chamber, protecting the eye from undesired complications such as choroidal effusion, suprachoroidal bleeding, and aqueous misdirection syndrome. In a study by de Barros et al., atropine eye drops were used therapeutically in patients with a flat anterior chamber [16]. Two other groups of patients underwent surgical revision, and the results were compared. As expected, surgical management compromised the vision of patients to a greater extent than did medical management. However, surgery was far more successful than atropine in terms of improving intraocular pressure.

As the benefits of atropine eye drops seem questionable, we wanted to examine the known side effects of atropine eye drops on patients’ self-reported visual quality. Concerning the overall assessment of visual quality, in the control group, there was no significant difference in the responses given two weeks after surgery in comparison to those given preoperatively. This was not the case for the patients who received atropine; two weeks after surgery, they reported significantly worse evaluations of overall visual quality compared with their preoperative assessments (Figure 1). Based on further patient responses, it was clear that their vision was impaired in many more specific qualitative aspects. Both study groups complained similarly of hazy vision, blurred vision, and difficulty focusing for 2 weeks after surgery. On the other hand, patients in the atropine group additionally complained of more frequent, severe and bothersome difficulty in estimating distances and depth and more frequent and bothersome light sensitivity and perception of haloes compared with their preoperative status. We see here a clear disadvantage caused by atropine eye drops in the early postoperative period: patients receiving atropine had an overall greater reduction in visual quality, as well as additional specific visual problems, compared with those who did not receive atropine.

In alignment with the obtained results, Hiraoka et al. reported a reduction in visual quality in children after only one week of cycloplegia through atropine eye drops [17]. A study by Li et al. also showed reduced visual quality in children receiving atropine over a concentration of 0.05% [18]. In further agreement with our results, another study using a similar questionnaire to assess visual complaints in schoolchildren receiving atropine showed disturbances concerning not only visual acuity but also visual functions such as accommodation, convergence ability, and stereopsis [19]. All the abovementioned studies targeted the influence of atropine eye drops on healthy subjects, whereas our study offers a first insight into patients suffering from known damage due to glaucoma and who have already undergone surgery

**Figure 3:**
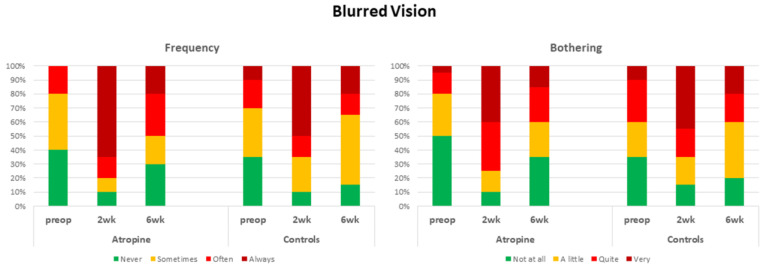
Frequency and bothersomeness of blurred vision reported by both groups. The percentage of patients given each possible value is presented. In both groups, there is an increase in the frequency and bothersomeness of blurred vision two weeks after surgery. Whereas these effects were corrected in the control group, in the atropine group, frequency and bothersome of blurred vision remained significantly high.

Six weeks after surgery, the overall visual quality in both groups was similar to the preoperative status (Figure 2). However, there were still differences concerning some qualitative aspects of vision. Whereas in the control group, all examined aspects were returned to the level of preoperative status, patients in the atropine group still reported more frequent and bothersome blurred vision (Figure 3) and starbursts. Thus, atropine eye drops slowed the physiological rehabilitation of vision in the late postoperative period.

In conclusion, atropine eye drops administered after surgery for their mydriatic and cycloplegic properties impair visual perception and prolong the time required for full rehabilitation. An important issue, as has also been shown by the usage of atropine in children for the prevention of myopia [20], is the concentration of the substance administered. It is possible that by using lower concentrations, we could achieve the desired protective effects of atropine in the anterior chamber after trabeculectomy while keeping visual impairment through its side effects to a minimum. Further studies on the anatomical and functional influence of lower concentrations of atropine on patients after glaucoma surgery would make it possible to define a more standardized postoperative management for them.

## Data Availability

Data of this study are available on demand from the corresponding author.

## References

[1] Shaarawy T. (2015). Glaucoma surgery: Taking the sub-conjunctival route. Middle East Afr. J. Ophthalmol..

[2] Razeghinejad M.R., Fudemberg S.J., Spaeth G.L. (2012). The changing conceptual basis of trabeculectomy: A review of past and current surgical techniques. Surv. Ophthalmol..

[3] Detry-Morel M., Pourjavan S., Detry M.B. (2006). Comparative safety profile between “modern” trabeculectomy and non-penetrationg deep sclerectomy. Bull. Soc. Belge Ophtalmol..

[4] Bakir B., Pasquale L.R. (2014). Causes and treatment of choroidal effusion after glaucoma surgery. Semin. Ophthalmol..

[5] Vaziri K., Schwartz S.G., Kishor K.S., Fortun J.A., Moshfeghi D.M., Moshfeghi A.A., Flynn H.W. (2015). Incidence of postoperative suprachoroidal hemorrhage after glaucoma filtration surgeries in the United States. Clin. Ophthalmol..

[6] Krix-Jachym K., Zarnowski T., Rekas M. (2017). Risk Factors of Malignant Glaucoma Occurrence after Glaucoma Surgery. J. Ophthalmol..

[7] Keenan J., Hakin J. (1992). Atropine after trabeculectomy. Ann. Ophthalmol..

[8] Pakravan M., Alvani A., Esfandiari H., Ghahari E., Yaseri M. (2017). Post-trabeculectomy ocular biometric changes. Clin. Exp. Optom..

[9] Mughannam A.J., Buyukmihci N.C., Kass P.H. (1999). Effect of topical atropine on intraocular pressure and pupil diameter in the normal horse eye. Vet. Ophthalmol..

[10] Joachimsen L., Farassat N., Bleul T., Bohringer D., Lagreze W.A., Reich M. (2021). Side effects of topical atropine 0.05% compared to 0.01% for myopia control in German school children: A pilot study. Int. Ophthalmol..

[11] Merli G.J., Weitz H., Martin J.H., McClay E.F., Adler A.G., Fellin F.M., Libonati M. (1986). Cardiac dysrhythmias associated with ophthalmic atropine. Arch. Intern. Med..

[12] Jimenez-Jimenez F.J., Alonso-Navarro H., Fernandez-Diaz A., Adeva-Bartolome M.T., Ruiz-Ezquerro J.J., Martin-Prieto M. (2006). Neurotoxic effects induced by the topical administration of cycloplegics. A case report and review of the literature. Rev. Neurol..

[13] Orengo-Nania S., El-Harazi S.M., Oram O., Feldman R.M., Chuang A.Z., Gross R.L. (2000). Effects of atropine on anterior chamber depth and anterior chamber inflammation after primary trabeculectomy. J. Glaucoma.

[14] Hoffmann E.M., Pfeiffer N. (2018). Trabeculectomy with mitomycin C: Video article. Ophthalmologe.

[15] McAlinden C., Pesudovs K., Moore J.E. (2010). The development of an instrument to measure quality of vision: The Quality of Vision (QoV) questionnaire. Invest. Ophthalmol. Vis. Sci..

[16] de Barros D.S., Navarro J.B., Mantravadi A.V., Siam G.A., Gheith M.E., Tittler E.H., Baez K.A., Martinez S.M., Spaeth G.L. (2009). The early flat anterior chamber after trabeculectomy: A randomized, prospective study of 3 methods of management. J. Glaucoma.

[17] Hiraoka T., Miyata K., Nakamura Y., Miyai T., Ogata M., Okamoto F., Oshika T. (2013). Influences of cycloplegia with topical atropine on ocular higher-order aberrations. Ophthalmology.

[18] Li W., Cao Y., Zhou J. (2022). Effects of low-concentration atropine eye drops on the optical quality of the eyes in myopic children. Indian J. Ophthalmol..

[19] Kuo H.Y., Ke C.H., Chen S.T., Sun H.Y. (2021). The Impact of Clinical Atropine Use in Taiwanese Schoolchildren: Changes in Physiological Characteristics and Visual Functions. Children.

[20] Ha A., Kim S.J., Shim S.R., Kim Y.K., Jung J.H. (2022). Efficacy and Safety of 8 Atropine Concentrations for Myopia Control in Children: A Network Meta-Analysis. Ophthalmology.

